# Identifying the roles of miR-17 in ciliogenesis and cell cycle

**DOI:** 10.3389/fcell.2024.1397931

**Published:** 2024-08-29

**Authors:** Ashwaq Alanazi, Ayan K. Barui, Ashraf M. Mohieldin, Ankan Gupta, Ramani Ramchandran, Surya M. Nauli

**Affiliations:** ^1^ Department of Biomedical and Pharmaceutical Sciences, Chapman University, Irvine, CA, United States; ^2^ Department of Pharmacology and Toxicology, Umm Al-Qura University, Makkah, Saudi Arabia; ^3^ Department of Pharmaceutical Sciences, California Northstate University, Elk Grove, CA, United States; ^4^ Department of Pediatrics, Division of Neonatology, Medical College of Wisconsin, Milwaukee, WI, United States

**Keywords:** MicroRNAs, primary cilia, cell proliferation, cell migration, PGRMC2, GM3S, PKD2

## Abstract

Emerging evidence suggests a significant contribution of primary cilia to cell division and proliferation. MicroRNAs, especially miR-17, contribute to cell cycle regulation and proliferation. Recent investigations have highlighted the dysregulated expression of miR-17 in various malignancies, underlining its potential role in cancer. However, the correlation between primary cilia and miR-17 has yet to be fully elucidated. The present study examines the presence of miR-17 in primary cilia. The miR-17 expression is studied in selected ciliary protein knockdown cells. Using *in situ* hybridization (ISH), we identified the subcellular localization of miR-17 in both cilium and cell body. We confirmed the importance of miR-17, progesterone receptor membrane component-2 (PGRMC2), and monosialodihexosylganglioside (GM3S) in cilia formation, as shown by the significant reduction in cilia and cilia length in knockdown cells compared to control. We also demonstrated the involvement of PGRMC2, GM3S, polycystin-2 (PKD2), and miR-17 in cellular proliferation and cell growth. Our studies revealed a hyperproliferative effect in the knockdown cells compared to control cells, suggesting the regulatory roles of PGRMC2/GM3S/PKD2/miR-17 in promoting cell proliferation. Overall, our studies conclude that ciliary proteins are involved in cell division and proliferation. We further hypothesize that primary cilia can serve as compartments to store and control genetic materials, further implicating their complex involvement in cellular processes.

## Graphical Abstract

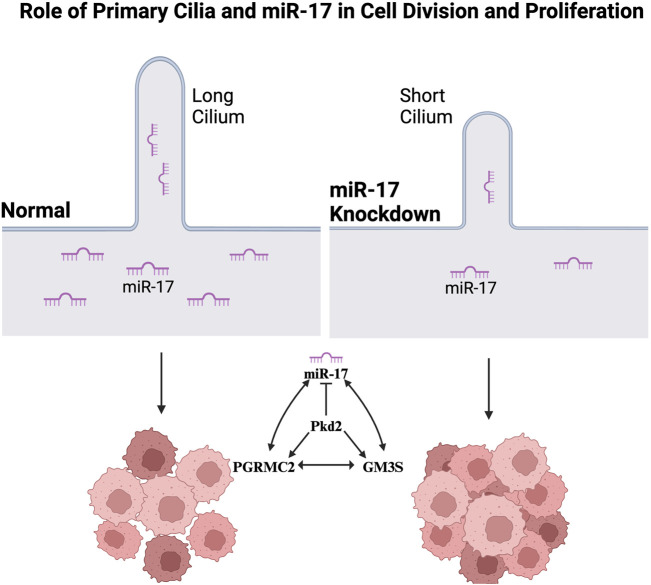

## 1 Introduction

The group of short non-coding RNA molecules (approximately 18–24 nucleotides in length) known as microRNAs (miRNAs) play a crucial role in both normal physiological and pathological conditions (Reinhart et al., 2000; Bartel, 2004). Substantial evidence supports the notion that miRNAs act as negative regulator of gene expressions by facilitating mRNA degradation and inhibiting protein translation (Bartel, 2004). Among the various miRNA clusters, the miRNA-17/92 (miR-17/92) cluster has emerged to have a significant role in development. Members of miR-17/92 cluster include miR-17, miR-18a, miR-19a, miR-20a, miR-19b, and miR-92a (Fang et al., 2017). The most prominent component of the miR-17/92 cluster is miR-17, which has a critical role in regulating most of the fundamental cellular functions such as cell cycle, cell proliferation, migration, and many others (Kong et al., 2019; Cloonan et al., 2008; Jiang et al., 2011).

The miR-17 can function as either oncogenes or tumor suppressors (Cloonan et al., 2008). Notably, miR-17 has been identified as an oncogene in cancer through extensive miRNome investigations (Lu et al., 2019; Volinia et al., 2006; Shan et al., 2009). These investigations demonstrate that miR-17 levels are significantly overexpressed in different types of solid tumors such as breast, lung, pancreas, colon, and prostate (Volinia et al., 2006). Conversely, miR-17 is identified as tumor-suppressive miRNAs *in vitro* and *in vivo*. The inhibition of miR-17 leads to upregulation of genes that are involved in the cell cycle (Sweat et al., 2021). Moreover, it is found that high serum levels of miR-17 decrease patients’ survival with lung cancer (Chen et al., 2012). These results collectively imply that the function of miR-17 in structures is associated with cell cycle regulation.

The primary cilia are hair-like organelles that are found on the surface of almost all mammalian cells. The ciliary membrane encloses the cilia, which the microtubule supports. The primary cilia principal function involves sensing and transmitting signaling pathways triggered by mechanical and chemical stimuli (Corbit et al., 2005; Praetorius and Spring 2003). These signaling pathways are critical in various cellular processes such as development, differentiation, and cell division (Singla and Reiter, 2006). Consequently, mutations that result in structural defects in primary cilia give rise to various diseases collectively known as ciliopathies (Lovera and Lüders, 2021; Ansley et al., 2003; Shao et al., 2020).

Ciliogenesis is a complex biological process in which the cilia are assembled and maintained on the surface of cells (Ishikawa and Marshall, 2011). The regulation of ciliogenesis is dynamically controlled during the cell cycle, with cilia being expressed and present during the G0 and G1 phases but undergoing resorption before entering mitosis (Plotnikova et al., 2009; Nigg and Stearns, 2011). This inverse relationship between primary cilia and cell proliferation is notable, as the primary cilium is reabsorbed into basal body (centrosome) during cell division, where the centrosome assumes the role of the mitotic pole (Goto et al., 2013). miR-17 is largely known to regulate cell cycle progression through several signaling proteins. miR-17 regulates E2F1, a critical regulator of cell cycle progression (Li et al., 2017; O’Donnell et al., 2005). miR-17 can downregulate expression of Cyclin D1, essential regulator of the G1 phase of the cell cycle (Karami et al., 2016; Sun et al., 2018). miR-17 targets and downregulates p21, affecting cell cycle control and promoting cell proliferation (Chen et al., 2016). miR-17 can also indirectly affect the RB1, a tumor suppressor protein that controls the cell cycle by inhibiting E2F transcription factors (Trompeter et al., 2011).

The relationship between cilia and miR-17 has been previously examined in ciliopathy polycystic kidney disease (Patel et al., 2013a; Noureddine et al., 2013). miR-17 targets and reduces the expression of KIF3A, which affects the assembly and function of cilia (Patel et al., 2013b). KIF3A is a component of the kinesin motor protein complex involved in the infraglabellar transport (IFT) necessary for ciliogenesis and ciliary maintenance. In addition, miR-17 has been shown to downregulate polycystin-1 (PKD1) and polycystin-2 (PKD2), crucial for the mechanosensory function of primary cilia in polycystic kidney disease (Lakhia et al., 2022; Nauli et al., 2003). Therefore, miR-17 may contribute to ciliary function and cyst formation.

Ultrastructural analysis of the ciliary bulb (or extracellular vesicle) reveals that miR-17 interacts with other ciliary proteins, including monosialodihexosylganglioside synthase (GM3S) and polycystin-2 (PKD2) (Mohieldin et al., 2015). In addition, some novel ciliary proteins have been recently identified. These cilia proteins include bone morphogenetic type 2 (BMPR2), transferrin receptor-1 (TfR1), junctional adhesion molecule-A (JAM-A), receptor protein tyrosine phosphatase sigma (PTPRS), and progesterone receptor membrane component-2 (PGRMC2) localized in the cilia (Mohieldin et al., 2015; Mohieldin et al., 2020). However, the mechanistic association between miR-17 and all these ciliary proteins is not currently known. Here, we examined if and how miR-17 interacted with all these proteins. We also studied miR-17 in cell division in relations with the ciliary proteins. We focused on the roles of miR-17 in cell division through primary cilia and its related mechanisms. Specifically, we investigated miR-17 knockdown epithelial cells and explored the proliferative effects and impact on cilia.

## 2 Materials and methods

### 2.1 Cell culture

Porcine renal epithelial cells (LLC-PK1; ATCC #CL-101) were cultured to a confluent monolayer in Dulbecco’s Modified Eagle Medium (DMEM) (Corning, catalog no. 10-013-CV) supplemented with 10% fetal bovine serum (FBS) and 1% penicillin-streptomycin solution (Cytiva HyClone™, catalog no. SH309100340) at 37°C in 5% CO2 incubator. We selected seven ciliary proteins to study: bone morphogenetic type 2 (BMPR2), junctional adhesion molecule-A (JAM-A/F11R), progesterone receptor membrane component-2 (PGRMC2), protein tyrosine phosphatase receptor sigma (PTPRS), transferrin receptor-1 (TfR1), monosialodihexosylganglioside (GM3S), and polycystin-2 (PKD2). Stable knockdown cells for each of the seven corresponding genes and scrambled control cells were previously generated in our laboratory (Mohieldin et al., 2015; Mohieldin et al., 2020).

### 2.2 *In situ* hybridization

The *in-situ* hybridization was used to identify the sub-cellular localization of miR-17. A miRCURY LNA™ Kit (Exiqon Inc. Qiagen, catalog no. 90000) was used for the *in-situ* hybridization targeting the microRNA miR-17. The Locked Nucleic Acid (LNA) probe for miR-17 was also obtained from Exiqon (Exiqon Inc. Qiagen, Design ID. 73515 cat no. 699990). The probe sequence was: 5′-TAC CTG CAC TGT AAG CAC TTT G-3’. The probe was tagged with Texas Red fluorescence TEX615. Cells were seeded into 18 × 18 coverslips (Fisher Scientific, catalog no. 12–542A) and incubated under normal growth conditions overnight until they reached 60%–70% confluency. Subsequently, the cells were then fixed for 10 min with 4% paraformaldehyde/2% sucrose in phosphate-buffered saline (PBS) (Corning, catalog no 21-040-CV) at room temperature, followed by permeabilization for 5 min with 10% triton X-100. Acetylated α-tubulin primary antibody (Sigma-Aldrich, catalog no. T6793) (1:10,000 dilution) was incubated with the cells overnight at 4°C for cilia visualization and staining, FITC conjugated anti-mouse secondary antibody (1:1000) was used. 10 μL of the probe was denatured at 80°C for 75 s. After hybridization of the slide in a humidified chamber for 30 min, the slide was then mounted with DAPI. We used a Nikon Eclipse Ti-E inverted microscope with NIS-Elements imaging software to capture images of primary cilia. To calculate the signal intensity of miR-17, we first identified the presence of cilia via acetylated-α-tubulin ciliary marker. A region of interest (ROI) was made based on the ciliary marker. This ROI was then transferred to the “miR-17” image. Signal intensity of this ROI was denoted as “cilium”. On the same “miR-17” image, cell boundary was drawn as our ROI and denoted as “total”. The intensity of miR-17 on the cell body was then calculated using the following formula (Equation 1).

cell body=total − cilium (1)

### 2.3 Isolation of primary cilia

After growing the cells in a 100-mm culture dish beyond confluence, the cells were washed briefly with 10 mL of PBS. The cell culture dishes were placed on MaxQ™ 2508 rotary shaker (Thermo Fisher Scientific, catalog no. CPA2480 3523-51) for 5 min at 350 rotations per minute (rpm). The PBS containing the isolated primary cilia was centrifuged at 4°C for 15 min at 1,000 × g using Legend X1R centrifuge (Beckman Coulter, Product No: B06320). The supernatant containing the isolated primary cilia was then transferred to a polyallomer tube and centrifuged for 30 min at 50,000 × g (16,700 rpm) at 4°C using WX+ 100 Ultra Centrifuge (Thermo Fisher Scientific, catalog no. 75000100).

### 2.4 Western blotting

The Western blotting was employed to confirm protein expression level. Following the preparation of a lysate from cell culture using lysis buffer supplemented with radioimmunoprecipitation (RIPA) buffer (Thermo Scientific, catalog no.89901) and protease inhibitor cocktail (100X) (Thermo Scientific, catalog no.1862209). The protein concentrations were determined using a Pierce™ bicinchoninic acid (BCA) protein assay kit from (Thermo Scientific, catalog no.23325). Afterward, 25 μg/μL of proteins were prepared using a 2x Laemmli sample buffer (Bio-Rad, catalog no. 1610737) and separated on 10% sodium dodecyl sulfate-polyacrylamide gel. The gels were run for 1–2 h at 100V. The protein samples were then transferred onto polyvinylidene difluoride membranes (Bio-Rad, catalog no. 10026934) using Trans-Blot^®^ Turbo™ machine (Bio-Rad, catalog no. 1704150). The membranes were blocked for 30 min with 5% non-fat dry milk (Bio-Rad, catalog no. 1706404) at room temperature. Subsequently, the blots were incubated at 4°C overnight) with primary antibodies, including PKD2-anti-mouse (Santa Cruz Biotechnology, catalog no. sc-28331) (diluted 1:1,000), GM3S-anti-rabbit (LSBio, catalog no. LS-C80886) (diluted 1:1,000), PGRMC2-anti-rabbit (Cohesion, catalog no. CPA2480) (diluted 1:1,000), GAPDH-anti-mouse (Santa Cruz, catalog no. sc-32233) (1:1,000), β-actin-anti-rabbit (Cell Signaling Technology, catalog no. 4970s) (diluted 1:1,000), and acetylated-α-tubulin-anti-mouse (Sigma-Aldrich, catalog no. T6793) (diluted 1:10,000). Finally, the membranes were covered with 1 mL of the chemiluminescent substrate (Thermo scientific, catalog no. 34580), and the signals were analyzed by ChemiDoc Touch Imaging System (Bio-Rad, model 1708370). For the transient re-expression studies, PGRMC2 was cloned into pCMV6-Entry vector to express a 223 amino acid protein (Origene, catalog no. rc204682).

### 2.5 Micro-RNA isolation

The miRCURYTM RNA Isolation Kit (Exiqon Inc. Qiagen, catalog no. 300110) was used according to the manufacturer’s instructions. The primary cilia pellets were lysed with 50 µL of lysis solution and then vortexed for 15 s. Subsequently, 50 µL of 96%–100% ethanol was added to the lysate and mixed by vortexing for 10 s. A total of 100 µL of the lysate with ethanol was applied onto the column provided with the kit and centrifuged for 1 min at >3,500 × g. The resulting flowthrough was discarded, and the spin column was reassembled with its collection tube. A volume of 100 µL of washing solution was added to the column and centrifuged for 1 min at 14,000 × g. This washing step was repeated two additional times. The column was spun for 2 min at 14,000 × g to ensure thorough drying of the resin. Subsequently, 50 µL of elution buffer was added to the column, and it was centrifuged for 2 min at 200 × g, followed by 1 min at 14,000 × g.

### 2.6 Quantitative PCR (qPCR)

The Universal Real Time microRNA PCR Kit was used according to the manufacturer’s instructions to perform the qPCR. For each template RNA sample, the concentration was adjusted to 5 ng/μL of total RNA. The reverse transcription reaction was prepared by mixing 2 μL of 5x Reaction buffer, 4.5 μL nuclease-free water, 1 μL enzyme mix, 0.5 μL synthetic RNA spike-ins, and 2 μL of template total RNA (5 ng/μL) to a total volume of 10 μL. Then, the reaction was mixed by vortexing and incubated according to the following protocol: 42°C for 60 min, followed by heat-inactivation of the reverse transcriptase for at 95°C for 5 min; then, it was immediately cooled to 4°C. After that, the cDNA was diluted 80 × with RNase-free water before quantification by qRT-PCR. For miRNA quantification the miRCURY LNA™ Universal RT micro-RNA PCR system (QIAGEN, catalog. no. 203450) was used in combination with pre-designed primer for miR-17 (QIAGEN, catalog. no. YP02119304). For each reaction, 5 μL master mix was added to 4 μL diluted cDNA template and 1 μL of PCR primer to a final volume of 10 μL. Expression of each micro-RNA was determined in duplicate at a final volume of 10 μL per well, using the CFX manager 3.1 Software (Bio-Rad). The reaction conditions for polymerase activation/denaturation were as follows: 95°C for 10 min, followed by 40 amplification cycles at 95°C for 10 s and 60°C for 1 min (ramp-rate 1.6°C/s). The qPCR was performed using the C1000 thermal cycler (Bio-Rad, Hercules, CA). Analysis and fold differences were determined using the comparative C_T_ method. The fold change was calculated from the ΔΔ*C*_T_ values with formula 2^−ΔΔ*CT*^.

### 2.7 RNAi knockdown

After seeding HEK-293T cells in each well of the 6-well plate to reach 50% confluency they were transfected with shRNA lentiviral vectors specific to miR-17 (pGFP-C-shLenti) (Origene, Cat. No. TR30021). Subsequently, the lentiviral particles were centrifuged at 4,000 rpm and passed through a 0.45 µm filter. Following this, cells were centrifuged at 2,500 rpm for 30 min with viral particles containing 8 μg/mL polybrene (Millipore, catalog no. TR-1003-G) and then cultured for up to 48 h, per manufacturer’s guidelines. Four targeting sequences were analyzed. RT-PCR confirmed knockdown efficiency of each sequence, and only one shRNA lentivirus with higher knockdown efficiency was selected. The shRNA sequences targeting miR-17 are listed in the table below.

**Table:** 

miR-17	Sequences
*A*	5′-GTG CAG GTA GTG ATA ATG TGC ATC TAC TG-3′
*B*	5′-GTT GCA CTA CAA GAA TGT AGT TGT GC-3′
*C*	5′-GCA GGA ATA AAG AGA CCA TCA CCT TGT AA-3′
*D*	5′-AGG CAC TTG TAG CAT TAT GGT GAC AGC TG-3′

### 2.8 Immunostaining studies

Cells were seeded on collagen-coated glass coverslips. Cells were then fixed in 4% paraformaldehyde (EMS, catalog no. 15710-SP-500) in PBS containing 2% sucrose (SIGMA, catalog no. T8787) for 10 min. Cells were then permeabilized in 10% Triton X (EMS, catalog no. 15710-SP-500). Next, cells were incubated overnight at 4°C with acetylated α-tubulin antibody (1:10,000 dilution) as a marker for primary cilia. After that, cells were incubated with fluorescence anti-mouse IgG secondary antibody (Vector Laboratories, catalog no. TI-2000) for 1 h at room temperature. Finally, the slide was mounted with HardSet™ Antifade Mounting Medium with DAPI (Vector laboratories, catalog no. 00158). The primary cilia images were acquired using Keyence Fluorescence Microscope at ×100 magnification, and their count and length were analyzed by the NIS-Elements software.

### 2.9 Cell cycle, proliferation, and cell growth

After growing the cells in culture dish under optimal conditions, cells were labeled with 30 µM BrdU (Millipore, catalog no. B5002) for 1 h at 37°C and 5% CO_2_. The subsequent steps involved the removal of BrdU media, a single rinse with PBS, and trypsinization to harvest the cells. Cells were fixed by resuspending the pellet in 0.3 mL PBS and gradually adding 0.7 mL ice-cold 100% EtOH. The mixture was gently agitated and stored at 4 °C for at least 1 h. Cells were incubated with 0.5 mL 2 N HCl/0.5% Triton X-100 for 30 min at room temperature to denature DNA. Afterward, cells were resuspended in 0.5 mL 0.1 M sodium tetraborate (Millipore, catalog no. B9876) for 2 min, followed by another round of centrifugation. After washing cells with 150 µL PBS with 1% BSA, the cells were resuspended in 50 μL PBS containing 0.5% Tween-20% and 1% BSA, followed by incubation with Alexa 488 conjugated BrdU antibody (Thermo Scientific, catalog no. B35130) for 1 h at room temperature in dark. The cells were pelleted again, then transferred to FACS tubes, and resuspended in 0.5 mL PBS containing 10 μg/mL RNase A and 20 μg/mL propidium iodide (PI) (BIOTIUM, catalog no. 40016). Samples were left at room temperature in the dark for 30 min before FACS analysis. Lastly, cells were analyzed with flow cytometry BDFacsverse. For each experiment 50,000 events were acquired at slow rate mode. Data acquisition was done by FACSDiva software and were subsequently analyzed by FlowJo software. For cell growth, the day before the experiment, a population of 1 × 10^5^ cells were plated in 6-well plate and provided with a growth medium. To determine the growth rate of cells, cell counts under light microscope were conducted daily until they reached >90% confluency. We observed that miR-17 knockdown cells exhibited minimal or no growth when cultured at a low cell density. It was noted that these cells required seeding at a higher density, impeding us from doing a proper cell growth assay.

### 2.10 Wound healing assay

The cells were prepared by seeding 1 × 10^5^ cells in 2 mL/well in 6-well plates, and the cells were incubated for 24–48 h. Upon achieving 90%–100% confluency, wounds were created by using a 200 μL micropipette tip. The culture medium was aspirated, and cells were washed with 1 mL PBS. Subsequently, 1 mL culture media containing 2% FBS was added to each well. Images were captured immediately after media replacement (T_0_) and every 24 h (T_24_), 48 h (T_48_), and 72 h (T_72_) period using Keyence Fluorescence Microscope at a ×4 magnification. Following image acquisition, the ImageJ wound healing plugin was used to determine the wound total area (μm^2^), the percentage of wound area (%), wound width (μm), and the standard deviation of the scratch width. In brief, by using a polygon selection tool to select the wound area, we were able to measure the wound area. We calculated the percentage of wound closure according to (Equation 1) at T_24_, T_48_, and T_72_. We calculated the rate of wound closure (μm/hr) according to (Equation 2).

$$Wound\;Closure\;\left(\%\right)=\left(\frac{\text{At}0-A\Delta t}{\text{At}0}\right)X100$$ (2)

A_t0_ is the wound area at time 0, and A_Δt_ is the wound area after n hours of the initial wound, both in μm^2^.

Rate of Wound Closure μm/hr=Wt0−Wt2424 (3)

W_t0_ is the wound width at time 0, and W_24_ is the wound width at time 24; both in μm.

### 2.11 Protein–protein interaction network

We used the StringApp plugin on Cytoscape software version 3.9.1 to analyze protein-protein interactions. Then, we loaded our proteins onto the StringApp from on our prior protein database (Mohieldin et al., 2020). We selected *homo sapiens* as the organism and confidence score threshold of 0.4. After that, the app used the STRING database to search for known and predicted protein-protein interactions based on our input data. StringApp generated a network of the protein-protein interactions, and then we further expanded the network to see all the possible interactions until we could find the interactions among proteins in our study. Then, the list of the protein interactions was further simplified by eliminating those proteins not closely or functionally associated with miR-17. For further confirmation, we loaded 16 proteins that interacted directly or indirectly with our proteins of interest, and they are as follows: PGRMC2, PKD2, GM3S, PTPRS, BMPR2, F11R, TMEM216, TUBA1A, ACTB, TFRC, ARL13B, FECH, UBC, NTRK1, UGCG, and TMEM231. Then, we added the interactions of these proteins with miR-17 based on our Western blot studies.

### 2.12 Data and statistic analyses

Cilia and wound healing images were acquired with Keyence Fluorescence Microscope. Images were analyzed with the NIS-Elements software. Cilia length and count were analyzed by NIS-Elements software. The number of independent experiments was indicated in each figure legend, and about 50 cells were quantified for each independent experiment. The wound distances and width closure were calculated with ImageJ software (version 1.53t). In our study, we used Prism 10 (GraphPad), Microsoft Excel (version 16.81) for statistical analysis and graphical representation. To ensure reliability of our results, each assay was performed in a minimum of three independent experiments. All data were presented as mean ± SEM. For the nonparametric and multiple comparison statistical analysis, we employed one-way ANOVA with *post hoc* Brown-Forsythe test followed by Tukey’s post-test. Statistical significance was considered as *p < 0.05, **p < 0.01, ***p < 0.001, ****p < 0.0001, or ns - not significant.

## 3 Results

We used porcine proximal epithelial cells LL-CPK1 (ATTC; # CL-101) as our experimental model, because our prior studies on miR-17 were previously performed in the same cell model (Mohieldin et al., 2015).

### 3.1 The miR-17 is localized to cilia and cell body

Using unbiased proteomic studies, we most recently discovered five new membrane proteins localized in the cilia: bone morphogenetic type 2 (BMPR2), transferrin receptor-1 (TfR1), junctional adhesion molecule-A (JAM-A), protein tyrosine phosphatase receptor sigma (PTPRS), and progesterone receptor membrane component-2 (PGRMC2) (Mohieldin et al., 2015; Mohieldin et al., 2020). We first screened if the subcellular localization or expression of miR-17 in the cilia was altered by any of these new ciliary proteins. We established knockdown cells for each of the targets and compared them to control cells for the subcellular localization of miR-17 using quantitative *in situ* hybridization (qISH) methodology. Efficacy for knockdown of the respective proteins were previously confirmed (Mohieldin et al., 2020).

**FIGURE 1:**
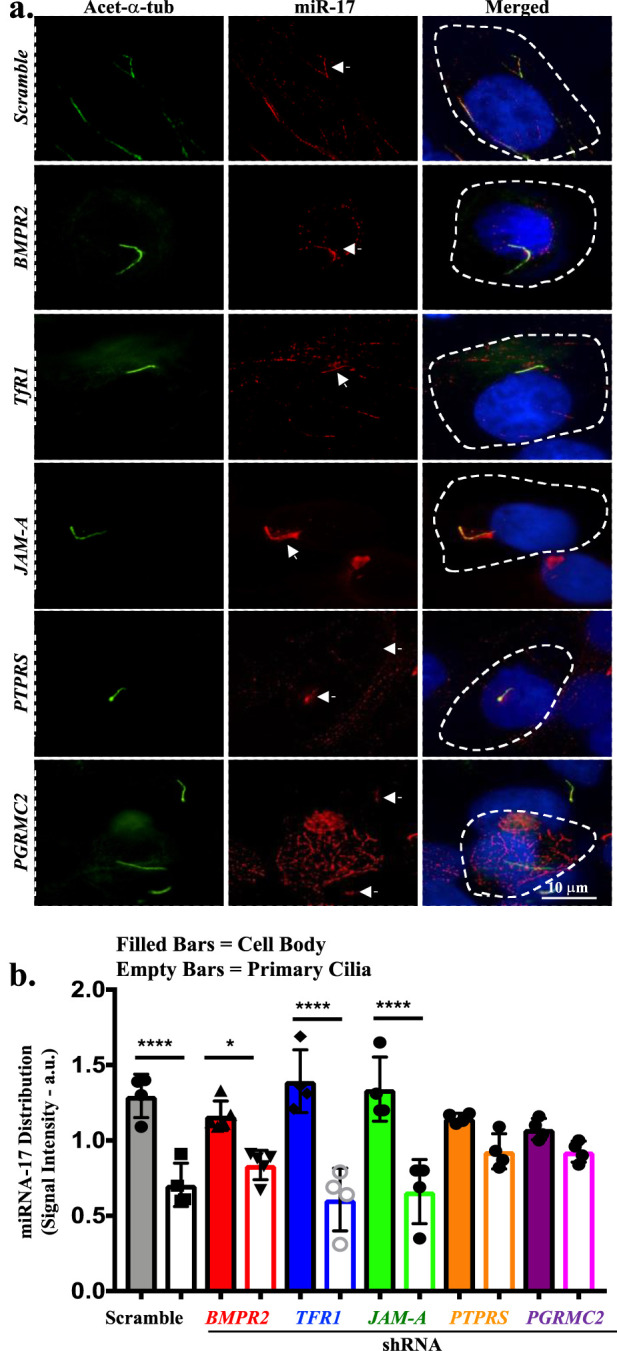
The miR-17 is localized to cilia and cell body. **(A)** The representative images of *in situ* hybridization show miR-17 localization in the primary cilia in scramble control, BMPR2 KD, TfR1 KD, PGRMC2 KD, JAM-A KD and PTPRS KD cells. The miR-17 localization was detected using locked nucleic acid probe for miR-17 (red); primary cilia were identified using acetylated-α-tubulin (green); DAPI was used as nuclear marker (blue). The miR-17 is localized in the cilia of all cells. **(B)** Quantitative the *in situ* hybridization assay was conducted to assess the levels of miR-17 between a cilium and a cell body within the same cell. The signal intensity was quantified in arbitrary units (a.u.). *p* < 0.05 (*); *p* < 0.0001 (****). N = 3 independent experiments.

The qISH was performed to identify the subcellular expression and location of miR-17. miR-17 expression levels in the cilia and cell body within the same cell were assessed (Figure 1A). The miR-17 LNA™ probe was tagged with red fluorescence TEX615, primary cilia were identified with acetylated α-tubulin, and the nucleus was stained with DAPI. In all BMPR2, TfR1, JAM-A, PTPRS, and PGRMC2 knockdown cells, including scrambled control cells, miR-17 were observed to co-localize with cilia. miR-17 was also observed in the cytosol of all cells. Of note is that the presence of miR-17 in the cytosol might also be attributed to the fact that the probe could recognize the immature pre-miRNA within the nucleus (Ha and Kim, 2014; Denli et al., 2004).

The qISH assay was conducted to assess the levels of miR-17 between a cilium and a cell body within the same cell (Figure 1B). The quantification involved measuring the intensities of fluorescence within a single cell. The intensity of the probe was varied among preparations, denoting that the probes might have different efficiency to hybridize with miR-17 in different knockdown cells. The quantitative *in situ* hybridization (qISH) revealed a significant higher of miR-17 expression in the cytosol than cilium by 1.91 ± 0.26 fold in scramble control cells (*p* < 0.0001). In BMPR2 knockdown cells, there was also a significant higher of miR-17 expression in the cytosol than cilium by 1.43 ± 0.15 fold (*p* < 0.05). Likewise, in TfR1 and JAM-A knockdown cells, there were significant higher of miR-17 expressions in the cytosol than cilium by 2.78 ± 0.93 and 2.50 ± 0.79 folds (*p* < 0.0001), respectively. On the other hand, there were no significant distributions of miR-17 between cytosol and cilium in PTPRS (1.35 ± 0.05 fold) and PGRMC2 (1.19 ± 0.10 fold) knockdown cells. Overall, these data indicate that miR-17 was localized in both cilia and cell body, indicating the significance of the role of miR-17 in the ciliary compartments.

### 3.2 The expression of miR-17 is decreased in PGRMC2 knockdown cells

**FIGURE 2:**
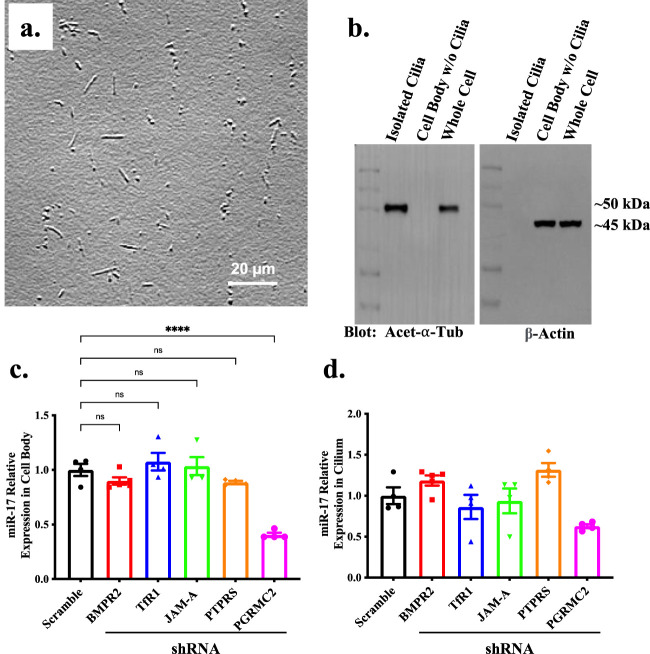
The expression of miR-17 is decreased in PGRMC2 KD cells. **(A)** To quantify miR-17 separately between cilia and cell body, cilia were isolated from the cells. Brightfield image displays the isolated cilia. **(B)** The purity of the isolated primary cilia was confirmed through immunoblotting, utilizing acetylated-α-tubulin (acet-α-tub) cilia marker and β-actin cell body marker. Un-cut original blots are shown in Supplementary Figure S1. RT-PCR was performed to determine levels of miR-17 in ciliary protein knockdown cells. The graphs represent the relative expression of miR-17 in cell body **(C)** and cilia **(D)**. *p* < 0.0001 (****). N = 4 independent experiments.

The qISH is a more appropriate method to compare miR-17 subcellular localization in a cell within the same preparations due to a potential variability in hybridization efficiency between different preparations. Because the qISH was not an ideal method to compare total miR-17 among different knockdown cells, we used quantitative PCR (qPCR) to measure a more precise expression of miR-17. To do this, we first isolated the primary cilia by using shear force to break the cilia of the cell surface (Mohieldin et al., 2015; Mitchell, 2013; Sherpa et al., 2019). We visually examined the fractions of the isolated cilia under the high resolution DIC microscope to evaluate the effectiveness and purity of our isolation (Figure 2A). The purity of the isolated cilia was also validated with Western blot analysis (Figure 2B). Cilia marker acetylated-α-tubulin and cytosolic marker β-actin were used to identify the purity of the cilia and cytosol (cell body) fractions.

We loaded the RNA templates for both cell body and cilia at a concentration of 5 ng/μL. Comparing control and different knockdown cells, we only observed a change of miR-17 expression in the cytosol of PGRMC2 knockdown cells (Figure 2C). The expression of miR-17 in the cytosol was significantly decreased in PGRMC2 knockdown, compared to control cells (0.52 ± 0.02 fold; *p* < 0.0001). There were no changes in miR-17 expression in the cilia between the control and knockdown cells (Figure 2D). Overall, these data indicate that cytosolic expression of miR-17 was decreased in the PGRMC2 knockdown, compared to the control cells.

### 3.3 Validation of PGRMC2, GM3S, and PKD2 knockdown

Previous studies have indicated that miR-17 has a molecular interaction with GM3S and PKD2 (Mohieldin et al., 2015). To investigate the mechanism of miR-17 repression, we probed the potential roles of GM3S and PKD2. The stable knockdown of PGRMC2, GM3S and PKD2 cells were previously generated in our laboratory (Mohieldin et al., 2015). To verify that the PGRMC2, GM3S, and PKD2 expressions remained repressed, we rigorously confirmed the integration of the shRNA reporter protein in the cells (Supplement Figure 2A). The expression of the reporter green fluorescence protein (GFP) was confirmed in each knockdown condition. We also validated the expressions of PGRMC2 (Supplement Figure 2B), GM3S (Supplement Figure 2C), and PKD2 (Supplement Figure 2D) using Western blots. The GAPDH and β-actin were used as loading controls. Analyses of the Western blots confirmed that the shRNAs significantly repressed the expression of PGRMC2 (26% ± 7%), GM3S (21% ± 10%), and PKD2 (5% ± 5%) (Supplement Figure 2E).

### 3.4 Cellular interactions of miR-17 with ciliary proteins

**FIGURE 3:**
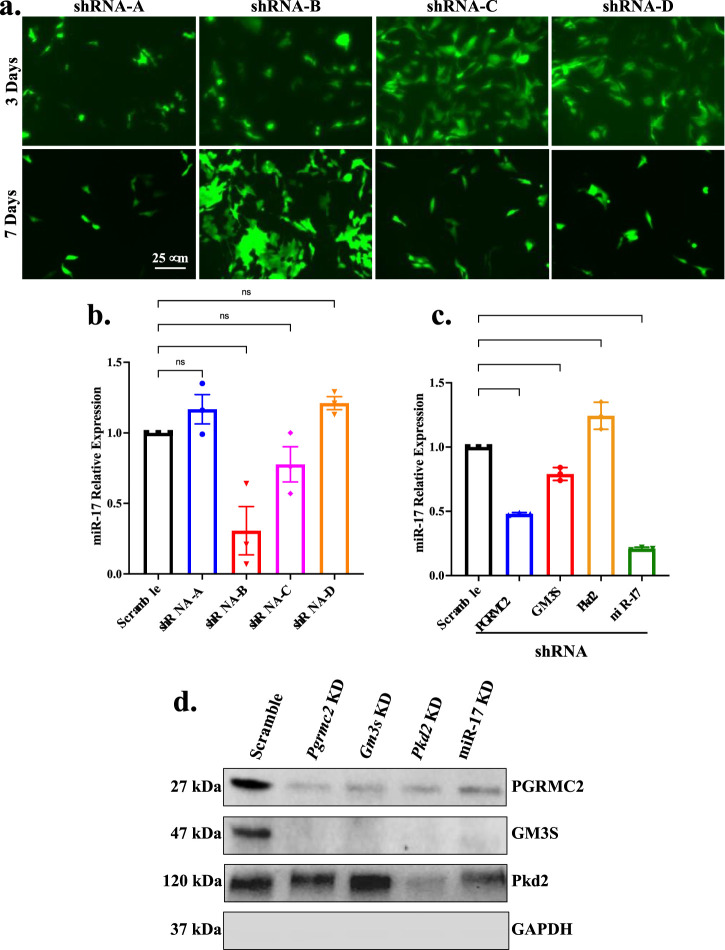
Cellular interactions of miR-17 with ciliary proteins. Stable miR-17 knockdown cell line was generated using siRNA. **(A)** Brightfield and fluorescence images displaying the effectiveness of transfection by tracking the expression of GFP in various shRNA-GFP lentivirus sequences (A, B, C, D). **(B)** Knockdown efficiency of lentivirus-mediated siRNA targeting miR-17 was determined by RT-PCR analysis. *p* < 0.01 (**); *p* < 0.0001 (****). N = 3 independent experiments. **(C)** Quantitative RT-PCR was performed to quantify the expression of miR-17 levels in PGRMC2, GM3S, and PKD2 knockdown cells. **(D)** Western blots were used to analyze protein expressions from the cell lysate in scramble control, PGRMC2, GM3S, PKD2, and miR-17 knockdown cells. Un-cut original blots are shown in Supplementary Figure S3.

To understand the significance of repressed miR-17 expression in PGRMC2 knockdown cells and miR-17 relevance in GM3S and PKD2 signaling, we also stably repressed the miR-17 expression with shRNA. Four different regions of silencing were used based on algorithm software prediction from OriGene company. The transfection efficiency of shRNA for each silencing sequence was determined through evaluating the expression of the GFP reporter integration in the cells (Figure 3A). Following transfection, transfected cells were cloned, subcloned, and cultured for several days. Subsequently, we selected the silencing sequence with the optimal knockdown efficiency among the four potential shRNA sequences, which was represented by positive GFP expression. The miR-17 knockdown was next quantified using RT-PCR analyses (Figure 3B). Among all other sequences, shRNA-B sequence significantly reduced the levels of miR-17. The levels of miR-17 transfected with shRNA-A, shRNA-C, and shRNA-D were found to have no significant effect. Thus, shRNA-B sequence was selected for our studies as the most effective sequence based on the GFP expression and lower miR-17 expression in qPCR analysis.

We next performed qPCR to quantify the expression of miR-17 levels in PGRMC2, GM3S, and PKD2 knockdown cells (Figure 3C). The expression of miR-17 was significantly higher in PKD2 knockdown cells compare to scramble control by 1.20 ± 0.06 fold (*p* < 0.01). However, PGRMC2 and GM3S repressed cells significantly reduced miR-17 expression levels compared to control by 0.50 ± 0.05 (*p* < 0.0001), and 0.80 ± 0.03 (*p* < 0.01) folds, respectively. As expected, silencing miR-17 repressed miR-17 expression by 0.21 ± 0.01 fold (*p* < 0.0001). These data suggested that PGRMC2, GM3S, and PKD2 regulated the miR-17 expression level. To further examine the intermolecular relationships among ciliary proteins PGRMC2, GM3S, PKD2 and miR-17, we analyzed the protein expressions from the cell lysate in scramble control, PGRMC2, GM3S, PKD2, and miR-17 knockdown cells (Figure 3D; Supplement Figure S3). Of note was that both PGRMC2 and GM3S expressions were repressed in all knockdown cells.

### 3.5 miR-17 is required for cilia formation

**FIGURE 4:**
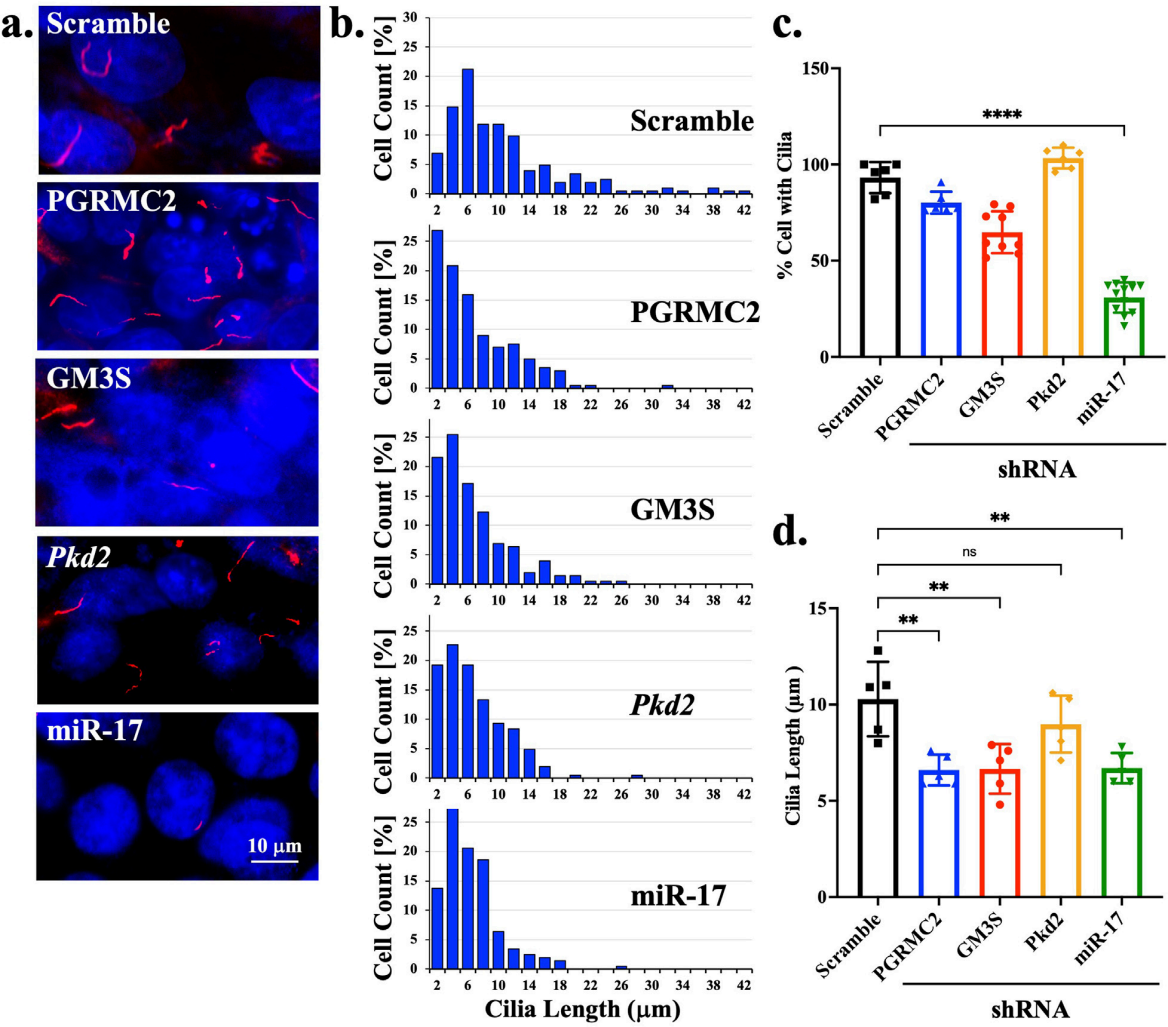
miR-17 is required for cilia formation. **(A)** The representative images show the cilia expression in scramble and different knockdown cells. The primary cilia were identified using acetylated-α-tubulin (red) and a DAPI was used as nuclear marker (blue). **(B)** Histogram shows the distribution of cilia length among different cells. The percentage of cells expressing cilia. **(C)** The graph shows the averaged of cells with cilia. **(D)** For those cells with cilia, the lengths of the cilia were measured. *p* < 0.01 (**); *p* < 0.0001 (****). N = 5 independent experiments.

Most ciliary molecules have been associated with cilia formation and length maintenance (Shao et al., 2020; Hua and Ferland, 2018; Pazour et al., 2000). To investigate the role of miR-17 on cilia structure, we also measured the potential roles of miR-17 cellular interacting components, including PGRMC2, GM3S and PKD2 on cilia presence and length. The presence of cilia was quantified with the cilia marker acetylated-α-tubulin, as seen in the representative images (Figure 4A). DAPI was used to label the nuclei. The ciliary length distribution was tabulated in the histograms (Figure 4B). The presence of cilia was then quantified. All knockdown cells expressed cilia, comparable to scramble control (Figure 4C). However, the miR-17 knockdown cells had a significantly lesser propensity to form cilia, with 30.8% ± 2.3% cells form cilia compared to control cells (93.1% ± 3.3%; *p* < 0.0001). If cells formed cilia, their cilia lengths were averaged (Figure 4D). Notably, there was no significant difference in the cilia length of PKD2 knockdown cells (8.9 ± 0.7 mm) compared to control (10.3 ± 0.9 μm). However, the cilia length was significantly decreased in PGRMC2 (6.6 ± 0.4 μm; *p* < 0.01), GM3S (6.7 ± 0.6 μm; *p* < 0.01), and miR-17 (6.7 ± 0.4 μm; *p* < 0.01) knockdown cells, compared to the control cells (10.3 ± 0.9 μm). Overall, these data suggest that miR-17, PGRMC2, and GM3S were required for cilia length maintenance.

### 3.6 Rescued miR-17 reverses the trend on primary cilia formation

**FIGURE 5:**
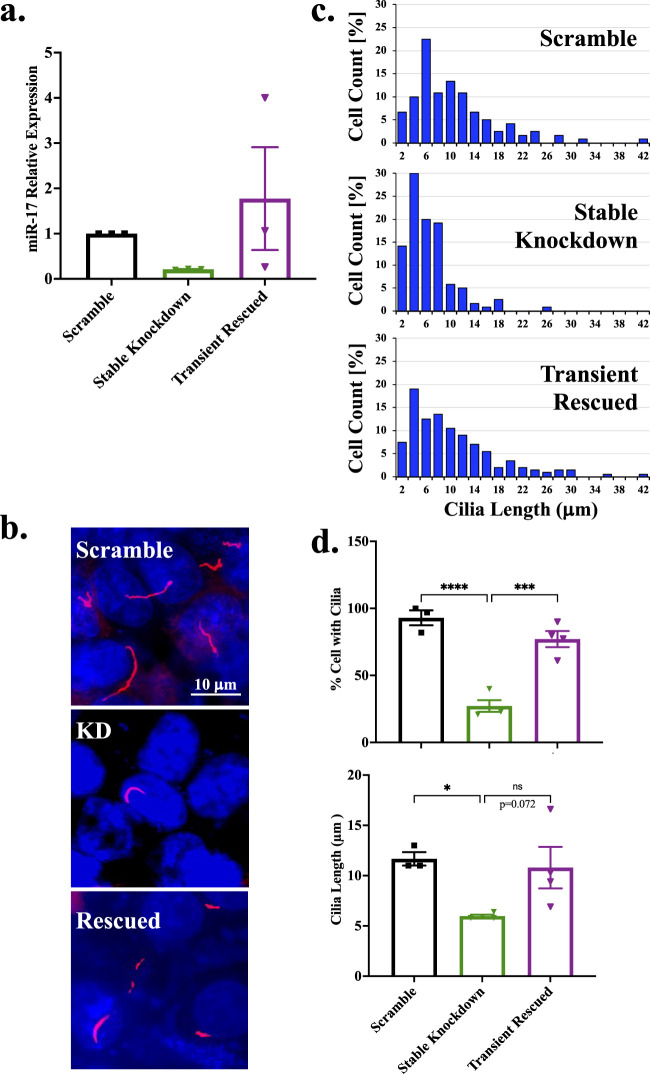
Rescued miR-17 reverses the trend on primary cilia formation. Stable miR-17 knockdown cell line was rescued with transient expression of anti-sense of shRNA miR-17. **(A)** Rescued efficiency of miR-17 was determined by RT-PCR analysis. N = 3 independent rescued experiments. **(B)** The representative images show the cilia expression in scramble, miR-17 knockdown (KD), and miR-17 rescue (rescued) cells. The primary cilia were identified using acetylated-α-tubulin (red) and a DAPI was used as nuclear marker (blue). **(C)** Histogram shows the distribution of cilia length among different cells. The percentage of cells expressing cilia. **(D)** The graph shows the averaged of cells with cilia. For those cells with cilia, the lengths of the cilia were measured. *p* < 0.05 (*); *p* < 0.001 (***); *p* < 0.0001 (****). N = 3-4 independent experiments.

To confirmed that the role of miR-17 on cilia formation and length maintenance, we performed a rescued experiment in the stably miR-17 knockdown cells. The rescue was done using transient approach via anti-sense of shRNA-B of miR-17. The transient miR-17 reversed the expression of miR-17 equivalent to the control (Figure 5A). The presence of cilia was quantified with the cilia marker acetylated-α-tubulin, as seen in the representative images (Figure 5B). The ciliary length distribution was tabulated in the histograms (Figure 5C). The presence and length of cilia were then quantified (Figure 5D). As expected, the miR-17 knockdown cells had a significantly lesser propensity to form cilia, with 27.2% ± 4.3% cells form cilia compared to control cells (93.0% ± 5.6%; *p* < 0.0001) and rescued cells (77.1% ± 6.0%; *p* < 0.001). While the lengths of cilia were not statistically different, the rescued miR-17 knockdown cells (10.8 ± 2.1 μm) showed a recovery trend of cilia length compared to the miR-17 knockdown cells (6.0 ± 0.6 μm; *p* < 0.07). The cilia length of the rescued miR-17 knockdown cells was almost equivalent to the length of the control cells (11.7 ± 0.7 μm). Overall, these data suggest that the role of miR-17 on cilia formation was reversible by the miR-17 expression level.

### 3.7 miR-17 and its interacting proteins are involved in cellular proliferation

**FIGURE 6:**
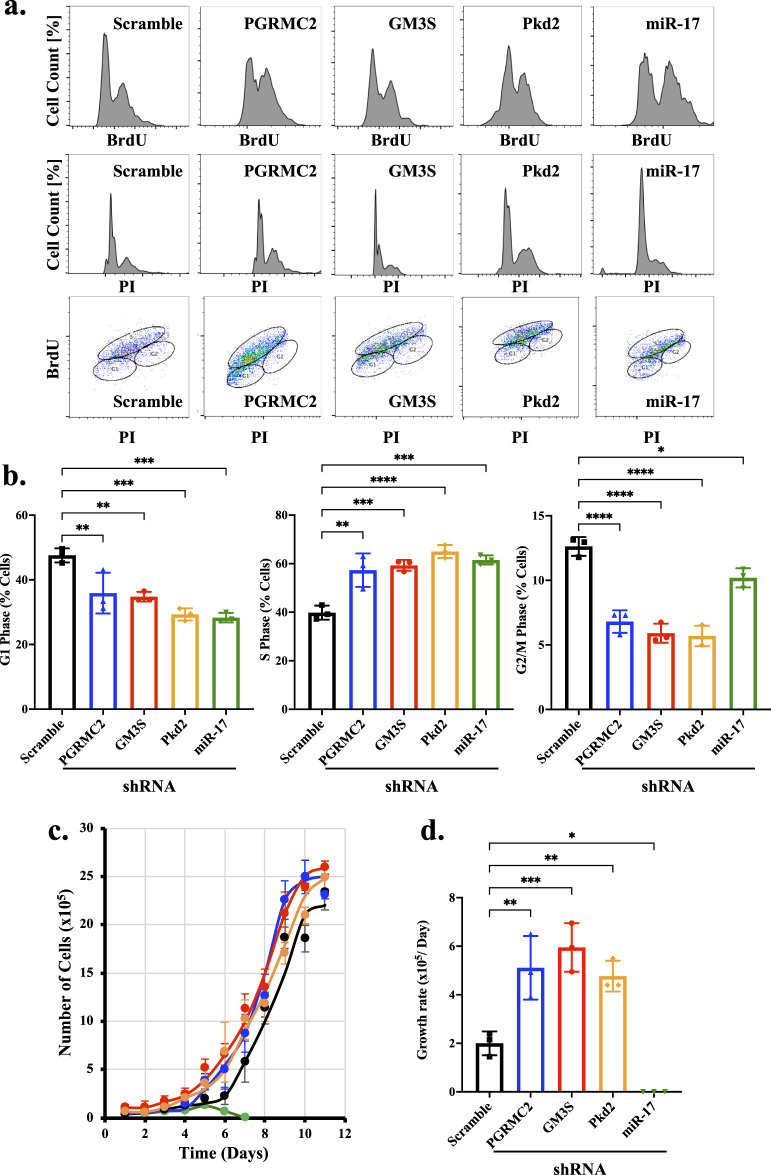
miR-17 and its interacting proteins are involved in cellular proliferation. Propidium iodide (PI) and 5-bromodeoxyuridine (BrdU) staining were used to examine cell proliferation. **(A)** Graphs depicting the percentage of cells with varying of PI and BrdU staining in each phase of the cell cycle are presented. **(B)** The graphs represent the percentage of cells in the G1, S, and G2/M phases of the cell cycle. **(C)** The number of cells was measured daily until cells reached complete confluence. **(D)** The growth rates were calculated. miR-17 KD cells did not grow when plated sparsely for the purpose of cell counts. *p* < 0.05 (*); *p* < 0.01 (**); *p* < 0.001 (***); *p* < 0.0001 (****). N = 3 independent experiments.

Most ciliary proteins are associated with cell proliferation (Shao et al., 2020; Wang et al., 2018). To investigate the role of miR-17 on proliferation, we measured the potential roles of miR-17, PGRMC2, GM3S and PKD2 using flow cytometry. Propidium iodide (PI) and 5-bromodeoxyuridine (BrdU) dual staining were used to analyze phases of the cell cycle (Figure 6A). PI denotes the DNA contents, and BrdU is to indicate actively dividing cells (Kinjyo et al., 2015; AbouAlaiwi et al., 2011). Analyses of the dual staining suggested that compared to control cells, all the knockdown cells had a statistically significant hyperproliferation as indicated in the resting G1 phase, proliferative S phase, and dividing G2/M phase (Figure 6B). Compared to control, the knockdown cells were significantly less at resting state, suggesting that knockdown cells were highly proliferative or undergoing cell division. This indicated that the hyperproliferative cells spent very little time in G1/G2/M phases, compared to S phase in synthesizing their DNA. Overall, these data suggest that PGRMC2, GM3S, PKD2, and miR-17 were involved in cellular proliferation.

To make sense of the cell cycle analyses that the hyperproliferative cells were perhaps lagging in S phase and rapidly progressing through G1/G2/M phases, we seeded 1 × 10^5^ cells and counted the cell numbers every day until the cells reached complete confluence (Figure 6C). The growth curves of the knockdown cells were then plotted against control cells. As expected from our cell cycle studies, the PGRMC2, GM3S, and PKD2knockdown cells displayed a significantly higher proliferation rate compared to the scramble control cells (Figure 6D). Notably, the growth rate was significantly slower in miR-17 knockdown cells compared to control cells. This was because miR-17 knockdown cells when cultured at low density of 1 × 10^5^ cells exhibited limited or no growth. However, these cells grew when seeding at 50% confluence, indicating that cell-cell interaction or communication might play a role in promoting their growth compared to when plated in very sparse conditions. Overall, these data suggest that PGRMC2, GM3S, PKD2, and miR-17 were involved in cell proliferation and growth.

### 3.8 miR-17 is involved in cell migration

**FIGURE 7:**
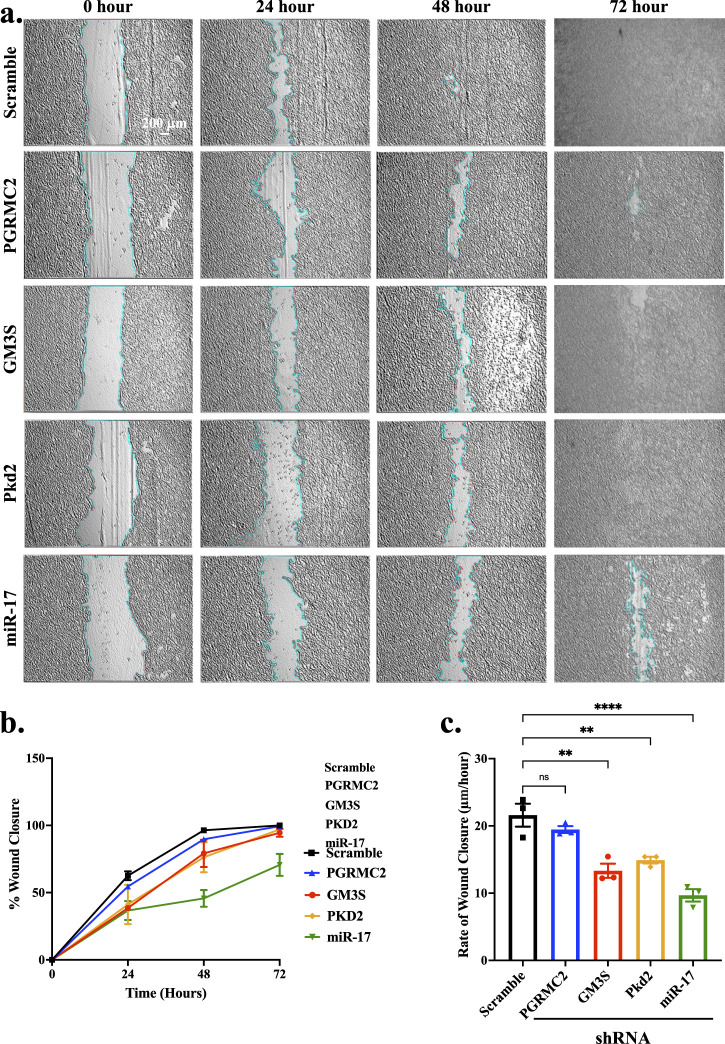
miR-17 and its interacting proteins are involved in cell migration. The wound healing assay was conducted to assess the cell migration. Wound-healing assay was performed at 0, 24, 48, and 72 h. **(A)** Representative images of the scratched and recovering of wounded areas (highlighted by light blue lines) on confluence monolayers. **(B)** The percentage of cell migration was determined by tracking the rate of cell movement towards the scratched area over time using ImageJ™ software. **(C)** The rate of wound closure per day was calculated. *p* < 0.01 (**); *p* < 0.0001 (****). N = 3 independent experiments.

Cell migration is a vital process in development, regeneration, and injury recovery (Todaro et al., 1965; Hossian and Mattheolabakis, 2021). Wound healing assay is one of convenient and straightforward techniques for investigating cell migration analysis. We performed cell migration assay to determine the functional impact of miR-17 and its interacting components PGRMC2, GM3S and PKD2. Monolayer cells were stretched accurately with an average width of 854.4 ± 15.6 µm (Figure 7A). The extent of cell recovering the wounded areas was measured at 24-h intervals (Figure 7B). After 72 h, the wound healing was closed or almost closed in control (100% ± 0%), PGRMC2 (99.35% ± 0.47%), GM3S (94.51% ± 2.9%), PKD2 (97% ± 0.89%), and miR-17 (70.63% ± 8.16%) knockdown cells. There was no statistically significant difference in wound healing closure between control and knockdown cells. We next measured and quantified the rate of wound closure (Figure 7C). There were no differences in the rate between control cells (21.6 ± 1.70 μm/h) and PGRMC2 knockdown cells (19.5 ± 0.51 μm/h). However, the migration rates were significantly lower in GM3S (13.3 ± 1.05 μm/h), PKD2 (14.9 ± 0.50 μm/h), and miR-17 (9.7 ± 0.92 μm/h) knockdown cells. Overall, these findings indicated a significant impact of miR-17 and its interacting components on the rate of cell migration.

### 3.9 Protein–protein interaction network

**FIGURE 8:**
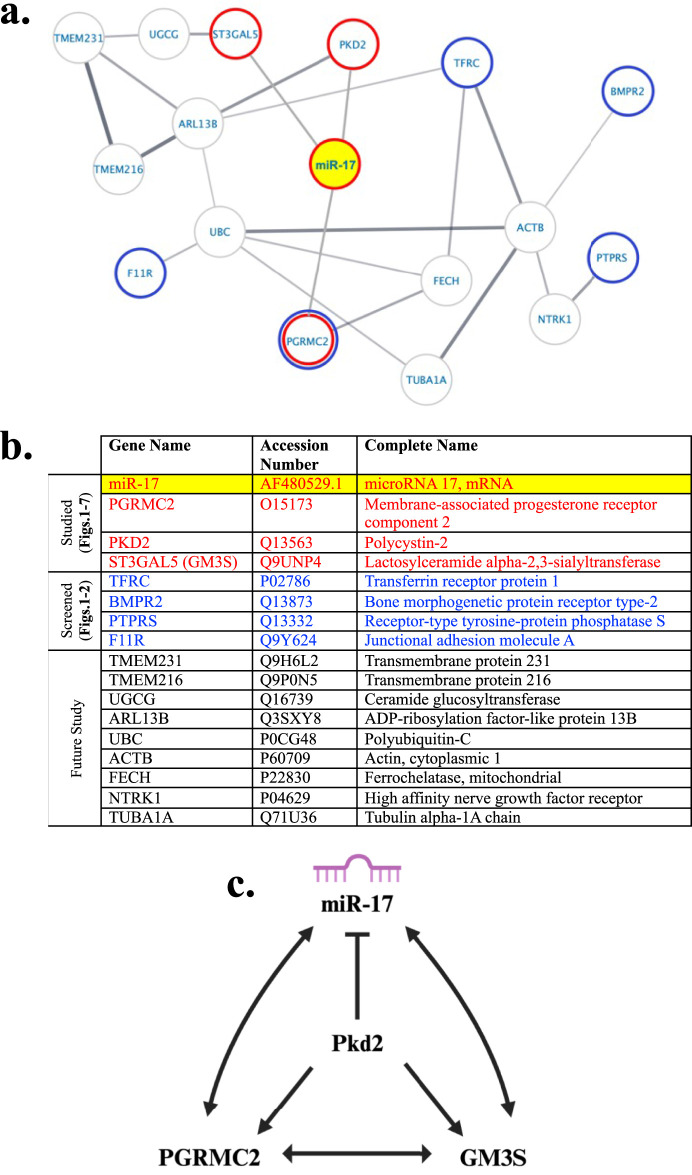
Potential ciliary protein interaction with miR-17. **(A)** Proteomic network analysis was performed using the Cytoscape StringApp tool for the analysis of potential protein-protein interactions taken from our prior cilia proteomic studies (Mohieldin et al., 2020). The network depicted interactions involving miR-17, highlighted in yellow, along with PGRMC2, GM3S, and PKD2, which are marked with a red border. Potential novel ciliary proteins examined with the *in-situ* hybridization in relationship with miR-17 include BMPR2, TfR1, JAM-A, PTPRS, and PGRMC2. These proteins are labeled with a blue border. **(B)** The Table listed the accession numbers of all proteins, including miR-17, with their complete names. **(C)** Our current studies suggested more specific molecular and functional interactions between miR-17 with PGRMC2, GM3S, and PKD2.

We conducted a protein-protein interaction (PPI) network analysis to explore potential interactions among the proteins identified in the previous and current studies (Mohieldin et al., 2020). The network was constructed using the StringApp, revealing predicted interactions with an average clustering coefficient of 0.54. The network comprised 16 nodes (proteins) connected by 19 edges (interactions), with an average node degree of 2.38 (Figure 8A). Additionally, the PPI enrichment *p*-value was notably low at 0.000412, indicating a highly significant functional relationship among the proteins within the network. It also indicated that the interactions detected in the network are not random. The PPI of individual proteins is listed in the Table showing their complete names and accession numbers (Figure 8B). The interaction among the ciliary molecules from our current studies was displayed and summarized in the diagram (Figure 8C). Overall, our Western blot analysis and functional studies supported the presence of intricate cellular interactions involving PGRMC2, GM3S, PKD2, and miR-17.

To further understand the complexity of the PPI network, we tested if an ectopic re-expression of the protein could rescue cilia and cell cycle phenotypes in miR-17 knockdown cells (Supplement Figure 4). We confirmed that miR-17 knockdown cells had a lower PGRMC2, and the PGRMC2 expression could be rescued by the PGRMC2 ectopic expression in the miR-17 knockdown cells (Supplement Figure 4A). These studies were repeated twice in the same blot with two independent sets of samples. We next checked cilia length and growth rate of cells (Supplement Figure 4B). The ectopic expression of PGRMC2 to a normal level did not rescue the cilia and cell cycle phenotypes in miR-17 knockdown cells. Because GM3S expression was also repressed in miR-17 knockdown cells (Supplement Figure 3), we examined GM3S expression level, which remained low in miR-17 knockdown cells with or without ectopic expression of PGRMC2 (Supplement Figure 4C). It is thus not surprising that ectopic expression of PGRMC2 alone in miR-17 knockdown cells did not rescue the cilia and cell cycle phenotypes, because miR-17 knockdown cells could potentially affect expressions of many other proteins in the network, in addition to PGRMC2, GM3S, or PKD2 (Figure 8).

## 4 Discussion

Our present study aimed at exploring the association between miR-17 and primary cilia, focusing on their roles in cell division and related mechanisms. The subcellular localization of miR-17 was investigated using *in situ* hybridization, revealing its presence in the cell body and cilia. For the first time, we showed that ciliary compartment carrying a genetic material miR-17. Furthermore, the qISH demonstrated that cytosol expressed higher miR-17 compared to the cilium. Knockdown of PGRMC2 resulted in depressed miR-17 expression. In addition to PGRMC2, our studies also demonstrated that miR-17 had cellular interactions with other ciliary proteins, including GM3S and PKD2. Our data demonstrated that miR-17 was involved in cilia structure, cellular proliferation, and cell migration. Our study also proposed a potential interaction between miR-17 and ciliary proteins, including PGRMC2, GM3S, and PKD2.

The subcellular localization of miR-17 within a cell could be attributed to the fact that subcellular compartments store and regulate genetic information (Putnam et al., 2023; Cheng et al., 2022). The observed decrease in miR-17 levels in PGRMC2 knockdown cells suggested that PGRMC2 played a critical role in regulating miR-17 expression. Moreover, PKD2knockdown increased miR-17 expression, suggesting potential negative feedback between miR-17 and PGRMC2, given that PKD2 could induce PGRMC2 expression. The cellular interactions among PGRMC2, GM3S, PKD2, and miR-17 were highlighting the complexity of their regulatory network within the cell. Our immunofluorescence studies revealed that miR-17 and its interacting proteins were involved in cilia formation, suggesting their importance for ciliogenesis and/or cilia maintenance. We observed an increase in proliferation in GM3S, PKD2, and miR-17 knockdown cells compared to control cells, while the cell migration assay showed a slower rate of wound closure in GM3S, PKD2, and miR-17 knockdown cells. This was because the relationship between cell proliferation and migration could be affected by several signaling pathways and cellular processes, such as GM3S/PKD2/miR-17 pathway. Moreover, the knockdown of ciliary proteins could modulate these pathways differently, resulting in contrasting effects in cell proliferation and migration.

In our experience, the qPCR method was not the best approach to compare miR-17 levels between cell body and cilia for two reasons. First, after isolation, the cell body and cilia-isolated lysates were normalized with two different proteins: β-actin for cell body and acetylated-α-tubulin for cilia (Figure 2B). Second, as indicated in our Method section, we loaded RNA templates for both cell body and cilia at a concentration of 5 ng/μL. It was obvious throughout our experiments that compared to cilia, cell body contained more total proteins and RNAs. For this reason, our initial comparisons between cilia and cell body were carried out using *in situ* approach, in which the cell and cilia were all treated and analyzed at the same conditions.

In certain cancer cell types, the loss of primary cilia was observed (Jamal et al., 2020). While cilia were expressed in normal kidney and prostate cancer cells (PC3), their absence was observed in both prostate cancer cells (DU145) and lung cancer epithelial cells (NL 20). Interestingly, while cilia were found in PC3, the percentage was significantly lower than that observed in normal kidney cells (Jamal et al., 2020). In our study, we measured the cilia length in PGRMC2 knockdown cells. The cilia length of PGRMC2 knockdown cells was significantly decreased compared to the control cells.

Previous studies showed a normal percentage of ciliated cells in PGRMC2 knockdown cells compared to control cells (Mohieldin et al., 2020), suggesting that there was a consistent effect on cilia presence. Consistent with the previous studies, our research also confirmed that knockdown of PKD2 led to a significant increase in proliferation compared to control. Likewise, our results were consistent with those reported by Jamal et al. (2020), who also observed hyper-proliferation in cells with PKD2 knockdown cells compared to control cells. This supported the proliferative role of PKD2 like those examined in our studies.

A previous study reported a significant decrease in the rate of wound closure in PGRMC2 knockdown cells compared to controls (Mohieldin et al., 2020). This discrepancy from our current study could arise from variations in experimental conditions between the two studies. Our study utilized cell culture media supplemented with 2% fetal bovine serum (FBS), while the previous study did not use any FBS. Serum starvation could induce cellular stress responses and change the cell behavior differently from cells subjected to media-containing serum. Serum media provided critical nutrients and growth factors that promoted cell proliferation and migration, while serum deprivation activated adaptive cellular responses that conserved energy to avoid nutrient deficiency. Serum-deprived cells might exhibit differing in migratory behaviors compared to those maintained in serum-containing media (Ahmadiankia et al., 2019; Raut et al., 2019).

Recent data confirm that miRNAs might also regulate several numbers of cell cycle regulators to regulate cell proliferation (Bueno and Malumbres, 2011). Dysregulation of miRNAs could contribute to proliferative disorders, including cancer, by altering the levels of critical oncogenes or tumor suppressor genes (Zhang et al., 2007). The use of miR-17 mimics promoted the proliferation through decreasing apoptosis *in vitro* (Song et al., 2020). Moreover, miR-17 expression levels were upregulated in the clinical samples from gastric cancer patients. The overexpression of PGRMC2 represses entry into the cell cycle (Yang et al., 2023), decreases the migration rates of cancer cells (Albrecht et al., 2012), and induces apoptosis (Yang et al., 2023). The overexpression of PKD2 rescues cells from stress-induced apoptosis (Brill et al., 2020). The miR-17 overexpression increases cell proliferation (Song et al., 2020; Brill et al., 2020). Of note is that when PKD2 was knockdown, we saw an increase in miR-17 expression (Figure 3C). Consistent with this idea (Song et al., 2020; Brill et al., 2020), we saw an increase in cell proliferation when miR-17 was overexpressed via PKD2 knockdown (Figure 6).

While our study showed significant effects of GM3S/PKD2/PGRMC2/miR-17 on ciliary dynamics and cellular proliferation, we did not explore a broader molecular pathway or signaling mechanism underlying these observations. Further investigation is warranted to fully understand the complex relationship among GM3S/PKD2/PGRMC2/miR-17 signaling, primary cilia, and cellular proliferation. Future studies should also explore potential correlations between primary cilia and other signaling pathways, such as the MAPK pathway and others, which were involved in cancer progression (Jamal et al., 2020). Elucidating these pathways and their interactions with primary cilia and miR-17 would provide better insights into the mechanism on how miR-17 regulated cell proliferation through primary cilia, leading to a better understanding of ciliopathies and the development of targeted therapeutic interventions.

We propose a potential pathway based on a network of ciliary proteins (Figure 8). While the network suggests a complex interaction among GM3S/PKD2/PGRMC2/miR-17, the Western blot confirmed the functional interactions among GM3S/PKD2/PGRMC2/miR-17, as shown in (Figure 3B). The miR-17 regulates expression levels of PGRMC2, GM3S, and PKD2. PKD2 suppresses miR-17 expression while increases the expression of PGRMC2 and GM3S. PGRMC2 elevates miR-17 and GM3S expressions but does not seem to influence PKD2 expression. Likewise, GM3S promotes miR-17 and PGRMC2 expressions without affecting PKD2. To sum up, the intermolecular relationship between miR-17 and the ciliary proteins PGRMC2, GM3S, and PKD2 involves a complex network of different regulatory interactions, which might involve microRNA-mediated post-transcriptional regulation and possible protein-protein interactions or signaling pathways. Further study is required to examine the exact mechanisms and functional effects of these interactions.

Remarkably, our studies demonstrated for the first time that the ciliary compartment carries a genetic material miR-17. This study provided novel insights into the complex relationship between miR-17, primary cilia, and cellular processes such as proliferation and migration. The findings suggested that miR-17 and ciliary proteins PGRMC2, GM3S, and PKD2 played crucial roles in regulating cilia dynamics and cellular behaviors.

## Data Availability

The original contributions presented in the study are included in the article/Supplementary Material, further inquiries can be directed to the corresponding author.
