# Supplementary material for: Identifying the roles of miR-17 in ciliogenesis and cell cycle
Source: Front Cell Dev Biol. 2024 Aug 29;12:1397931. doi: 10.3389/fcell.2024.1397931 (PMC11390542; doi:10.3389/fcell.2024.1397931)
Supplement: Supplementary file 1 [file DataSheet1.pdf]

*Supplementary Material*

# Identifying the Roles of miR-17 in Ciliogenesis and Cell Cycle

**Ashwaq Alanazi[1,2], Ayan K. Barui[1], Ashraf M. Mohieldin[3], Ankan Gupta[4], Ramani Ramchandran[4], Surya M. Nauli[1]**

[1]Department of Biomedical and Pharmaceutical Sciences, Chapman University, Irvine, CA, USA

[2]Department of Pharmacology and Toxicology, Umm Al-Qura University, Makkah, Saudi Arabia

[3]Department of Pharmaceutical Sciences, California Northstate University, Elk Grove, CA, USA

[4] Department of Pediatrics, Division of Neonatology, Medical College of Wisconsin, Milwaukee, WI, USA

**Correspondence:**
Surya M. Nauli
nauli@chapman.edu

## Supplement Fig.1

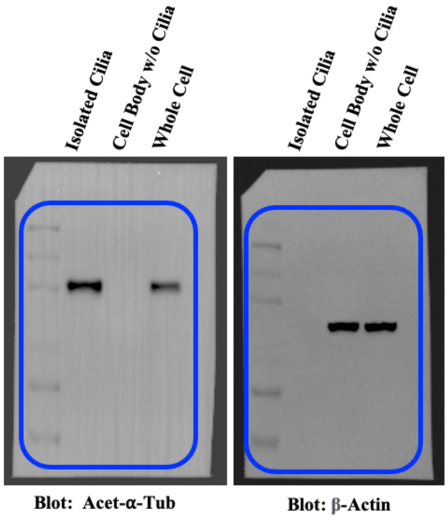


**<u>Supplement Figure 1. Original uncropped Western blots.</u>**

The original un-cut Western blots are shown. These blots were used in Figure.2b.

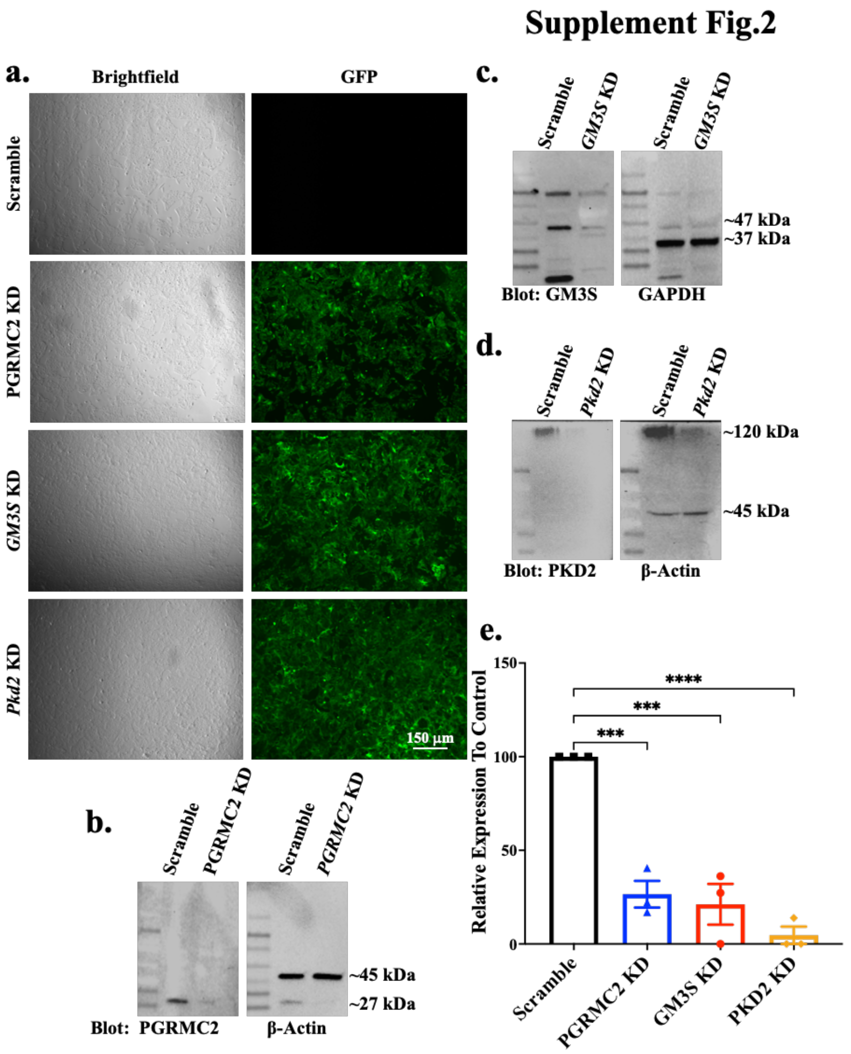


**Supplement Figure 2. Knockdown validation of PGRMC2, GM3S, and PKD2.**

**(a)** The effectiveness of transfection in PGRMC2, GM3S, and PKD2 knockdown cell lines was determined by assessing the expression of the reporter protein, green fluorescence protein (GFP). Additionally, cell lysates from scramble, PGRMC2, GM3S, and PKD2 knockdown cells were examined to further confirm the efficiency of the knockdown process. To validate the knockdown efficiency for PGRMC2 (**b**), GM3S (**c**), and PKD2 (**d**), we employed the Western blot technique. GAPDH and β-actin were utilized as loading controls. **(e)** Relative protein expression was quantified. $p<0.001$ (***); $p<0.0001$ (****). N=3 independent experiments.

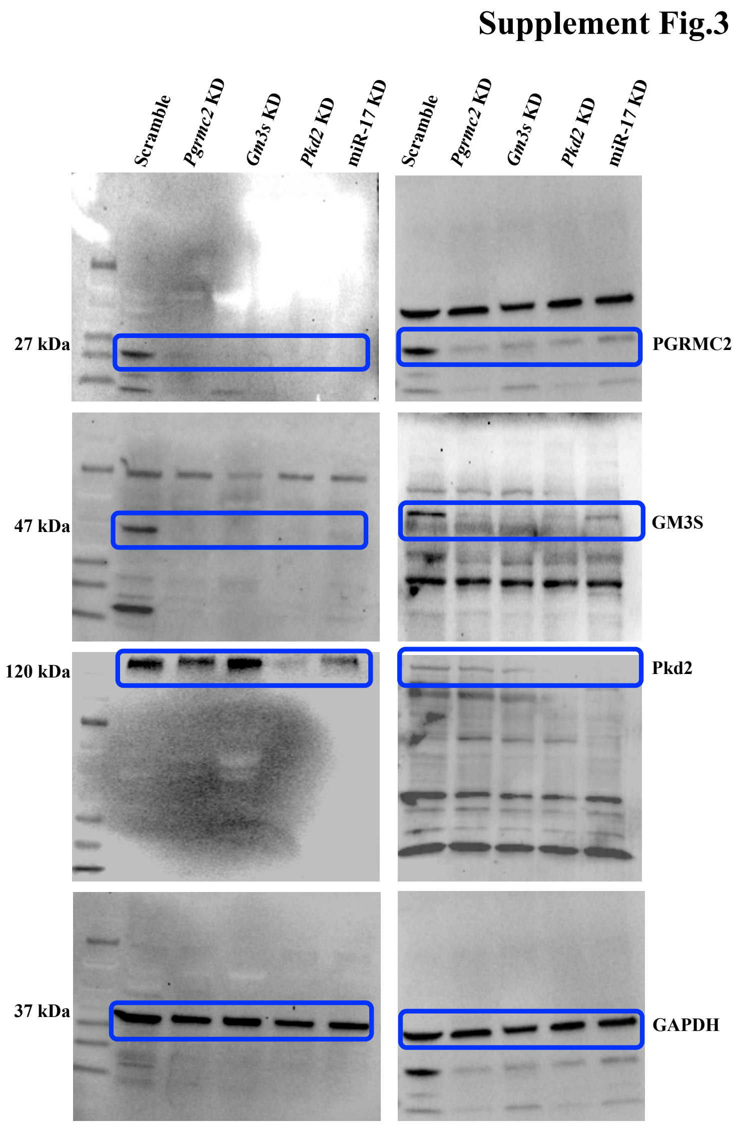


**Supplement Figure 3. Original uncropped Western blots.**

The original un-cut Western blots are shown. The left set of blots were used in Figure.3d. The two sets of the blots were carried out using different batches of the antibodies. The samples were also generated independently between the two sets of blots.

**Supplement Fig.4**

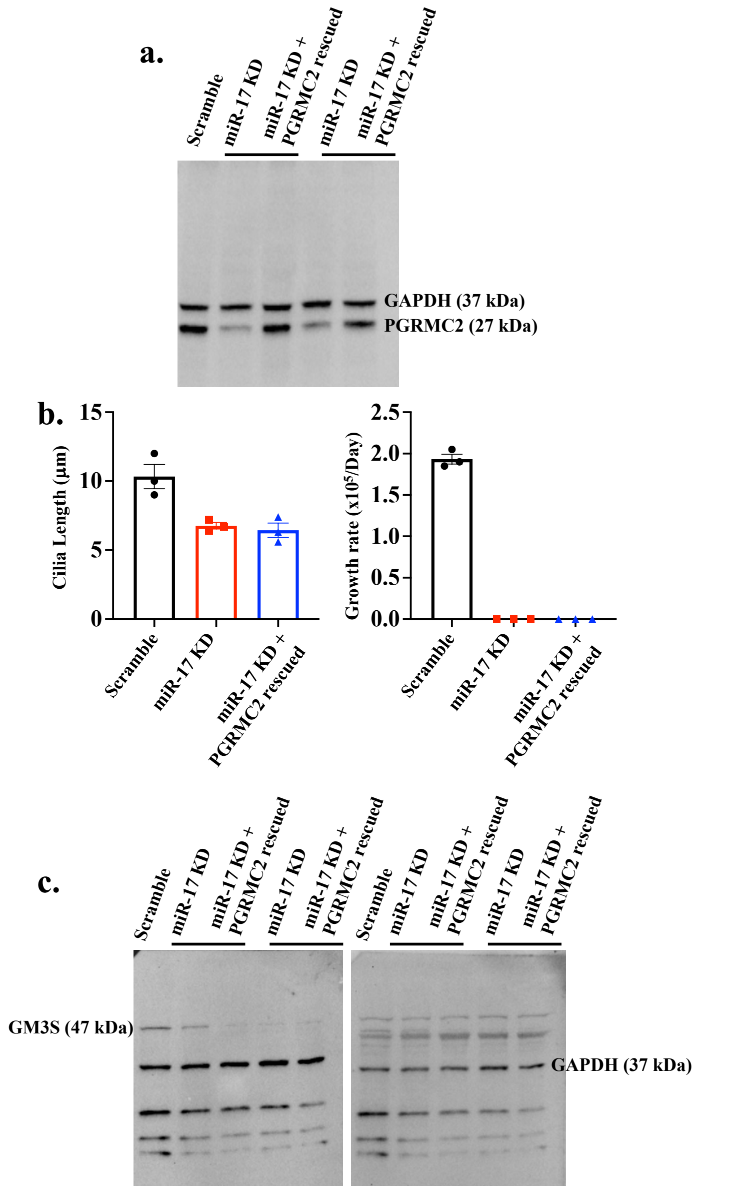


<u>**Supplement Figure 4. Ectopic expression of PGRMC2.**</u>

**a.** The miR-17 stable knockdown cells (miR-17 KD) had a lower PGRMC2, and the PGRMC2 ectopic expression was induced in the miR-17 KD cells (miR-17 KD + PGRMC2 rescued). These studies were repeated twice in the same blot with 2 independent sets of samples. **b.** cilia length and growth rate of cells were quantified with and without PGRMC2 rescued in miR-17 KD cells. **c.** GM3S expression levels were examined low in miR-17 KD cells with and without PGRMC2 rescued. GAPDH was first blotted to ensure equal loading; membrane was then stripped and reblotted for GM3S.
